# Internet of Things Geosensor Network for Cost-Effective Landslide Early Warning Systems

**DOI:** 10.3390/s21082609

**Published:** 2021-04-08

**Authors:** Moritz Gamperl, John Singer, Kurosch Thuro

**Affiliations:** 1 Chair of Engineering Geology, Technical University of Munich, 82024 Munich, Germany; thuro@tum.de; 2 AlpGeorisk, 85716 Unterschleißheim, Germany; singer@alpgeorisk.de

**Keywords:** early warning system, landslides, geosensors, monitoring, Colombian Andes, low income settlements, informal settlements, IoT

## Abstract

Worldwide, cities with mountainous areas struggle with an increasing landslide risk as a consequence of global warming and population growth, especially in low-income informal settlements. Landslide Early Warning Systems (LEWS) are an effective measure to quickly reduce these risks until long-term risk mitigation measures can be realized. To date however, LEWS have only rarely been implemented in informal settlements due to their high costs and complex operation. Based on modern Internet of Things (IoT) technologies such as micro-electro-mechanical systems (MEMS) sensors and the LoRa (Long Range) communication protocol, the Inform@Risk research project is developing a cost-effective geosensor network specifically designed for use in a LEWS for informal settlements. It is currently being implemented in an informal settlement in the outskirts of Medellin, Colombia for the first time. The system, whose hardware and firmware is open source and can be replicated freely, consists of versatile LoRa sensor nodes which have a set of MEMS sensors (e.g., tilt sensor) on board and can be connected to various different sensors including a newly developed low cost subsurface sensor probe for the detection of ground movements and groundwater level measurements. Complemented with further innovative measurement systems such as the Continuous Shear Monitor (CSM) and a flexible data management and analysis system, the newly developed LEWS offers a good benefit-cost ratio and in the future can hopefully find application in other parts of the world.

## 1. Project Overview Inform@Risk

### 1.1. Project Background and Goals

Landslide hazard is increasing year by year due to more intense and frequent rainfall as a consequence of climate change [1]. Additionally, secondary causes such as wildfires play an important role in promoting landslides [2]. The poorest residents are often the most vulnerable to landslides because in many cases they live in the most dangerous areas [3,4,5,6]. This is especially true in the Andes, where more than 10 million people are exposed to natural hazards and a high degree of inequality is prevalent [7,8]. In Colombia, recent developments have led to an increased rural depopulation and migration into cities, leading to an exponential increase in landslide casualties in the last 50 years [9], with more than half a million families affected by landslides in the past 100 years [9,10]. Rainfall is the most important triggering mechanism for these landslides, although human interference also is an important trigger [11,12].

The research project Inform@Risk aims to strengthen the resilience of informal settlements against rainfall-induced landslides. Since long term risk reduction solutions, as, e.g., the relocation of the endangered inhabitants or the implementation of physical mitigation measures, are currently not feasible due to the high social, political and monetary efforts required and their potential to harm the landscape and ecosystem [13], the Inform@Risk project is designing and implementing a Landslide Early Warning System (LEWS) as a short- to medium-term risk reduction solution. In order to ensure the successful implementation of such a system, many different technical and social challenges have to be overcome. The main challenges are the design of a cost-effective, reliable and spatially and temporally highly resolved monitoring system and its social integration into the informal settlement where it is implemented. The system can not succeed if it is not supported and trusted by the local residents it is designed for. Adequate risk perception and knowledge of the population is another important factor that determines whether the system can make a lasting impact [14]. Overall, the system needs to address the following points to be successful. It needs to be:

1. Socially integrated;
2. Spatially integrated;
3. Multiscalar;
4. Multisectoral;
5. Precise;
6. Inexpensive;
7. Easily replicable.

This requires intensive interdisciplinary cooperation between scientists, governmental and social institutions as well as the local communities. Therefore, a “living lab” approach is pursued, in which all measures, from geological investigations and the layout of the early warning system to the testing and training phase, are intensively discussed and consulted with the local residents.

To address this, the German team of the Inform@Risk project includes landscape architects (Leibniz University Hannover, LUH), geologists (Technical University of Munich, TUM), geotechnical instrumentation experts (AlpGeorisk, AGR), remote sensing experts (German Aerospace Center Oberpfaffenhofen, DLR and Expert Office for Aerial Image Evaluation and Environmental Issues, SLU), and software developers (Technical University Deggendorf, THD). The Colombian team is comprised of representatives from the center of urban studies (EAFIT university, Medellin) and several municipal agencies responsible for risk management in the city of Medellín (e.g., SIATA, DAGRD) as well as two local NGOs and the Colombian Geological Society.

In the following, after shortly introducing the study area, the technical design of the proposed LEWS is presented, focusing on the newly developed wireless geosensor network. Other essential elements of the research project, for example the landslide hazard and risk assessment and the social work are only covered superficially and will be published in detail elsewhere. A list of publications related to the Inform@Risk research project can be found at https://www.bmbf-client.de/projekte/informrisk (accessed on 1 March 2021).

At the time of writing, the Inform@Risk project has reached about two thirds of its 3-year duration (2019–2022) with the finalization of the design of the LEWS. The implementation of the system has been delayed due to the COVID-19 crisis and is currently scheduled for summer/fall 2021.

### 1.2. Project Area

In Medellín, the second largest Colombian city, landslides are the most common and deadly natural hazard [15]. Compared to the rest of the larger region (Valle de Aburrá), Medellín has by far the highest number of landslide reports [16]. Especially the steep slopes in the east of the city are prone to landslides [17,18]. The four deadliest landslides in Medellín in the last 100 years took place in this area, where at the same time informal settlements have shown some of the highest growth rates in the city. This lead to a continuously increasing landslide risk and heavy losses of life in the past 100 years. Based on a 2012 study, more than 200,000 inhabitants in the Aburrá Valley are subject to medium or high landslide risk and live in precarious settlements [17].

This is also where the barrio “Bello Oriente” (Communa 3) is located, which was selected as a test site for the early warning system of the Inform@Risk project. The informal settlement lies on the urban-rural border of the city and was chosen based on a preliminary qualitative risk assessment at city scale as well as additional social factors (security, community management etc.).

### 1.3. Geology and Landslide Processes

The geology of Medellín is dominated by magmatic and metamorphic rocks, as well as landslide debris and alluvial deposits. In the east of the city, amphibolites and dunites are the predominant rocks. The Medellín Dunite is primarily serpentinized and highly fractured due to tectonic processes. This leads to complex weathering profiles, which makes the dunite particularly prone to landslides [19]. Geophysical investigations have shown that the weathering depth can reach up to 50 m in some areas [20]. On top of the bedrock, residual soil (saprolite) alternates with slope deposits (colluvium) of varying depth. While the saprolite is usually clay dominated and free of stones and blocks near the surface, the composition of the slope deposits can vary greatly. It can best be described as “block in matrix” material, where many stones and blocks are embedded in a clay dominated matrix. Overall, the soil profile on top of the weathered dunite is very complex and heterogeneous.

As geological and morphological mapping of the project area has shown, the prevalent landslide process is rotational sliding, followed by a comparably small number of translational slides, debris flows in the creeks and minor rockfall at the few rock outcrops within the project area [18]. The slides usually are of small to medium size and depth (up to about 50 m in length and width, and up to 10 m deep). Due to the geological heterogeneity of the subsurface, it is impossible to predict exactly where future slides within the soil cover might occur. However, mainly based on the depth of soil cover (colluvium and saprolite) and the slope inclination it is possible to spatially differentiate the level of landslide hazard throughout the project area. Regarding the velocities of the landslides, we expect initial slow to moderate movements, which can accelerate to rapid and very rapid movements, depending on the water saturation [21].

## 2. Landslide Early Warning Systems

Since their origin in the 1980s, numerous Landslide Early Warning Systems (LEWS) have been designed and implemented. Some of them focus on a regional, national or global scale (so called “territorial systems”), while other systems focus only on a single slope or landslide (local LEWS). Guzzetti et al. (2020) and Piciullo et al. (2018) highlight a number of territorial systems and present the challenges and possibilities that are inherent to LEWS of this scale [22,23]. Because of the scale, for these types of LEWS only a generalized warning for a region is possible, while localized early warning of a specific area or slope is not possible.

Local LEWS usually include multiple sensors on site, cover a much smaller area (<0.1 km${}^{2}$ to 1 km${}^{2}$) [24] and, most importantly, usually are installed in areas where slow movements have already been detected and thus the exact location of a potential landslide is already known. Pecoraro et al. (2019) reviewed several local LEWS around the world and found that the primary parameter used for warning is displacement, followed by the trigger—mostly rainfall—and groundwater parameters. This makes sense since rainfall is the most important triggering factor for landslide early warning [25]. By Pecoraro et al. (2019), also a comparison of the sensor systems in use is made, with the most important monitoring systems being classical geotechnical methods such as inclinometers and piezometers, as well as geophysical (geophones), geodetic (GPS) and remote sensing methods. Yet, such studies rarely take costs into account, which is one of the most important parameters when working in low-income countries [24,26]. Low-Cost monitoring systems until now are mostly limited to GPS networks, acoustic emissions or radio communication [27,28,29,30,31].

### 2.1. LEWS in Medellín and Colombia

In Colombia, a LEWS on national scale already exists by “IDEAM”—Instituto de Hidrología, Meteorología y Estudios Ambientales [32]. Another system focussing on debris flows exists in Combeima-Tolima, which combines rainfall and geophone data on a regional scale to react once the dynamic thresholds are reached [14,33,34]. On the municipal level, LEWS exist in Bogotà and Medellín (www.idiger.gov.co) (accessed on 1 March 2021), although most of them are regional systems. There are local LEWS in Colombia that focus on landslides, for example a system in the city of Bolivar, based on an open-source system which measures humidity and temperature to determine the risk of landslides [35]. Yet, site-specific warning systems, especially with community involvement and evacuation alarms, are still rare in urban regions [36].

### 2.2. The Internet of Things and Landslide Monitoring

In the last decade, the Internet of Things (IoT) has become an important concept for landslide monitoring. Its definition, “Sensors and actuators embedded in physical objects are linked through wired and wireless networks” [37], describes why the concept is well applicable to landslide monitoring and early warning. The IoT can help circumvent the problem of often very expensive monitoring systems and other possible problems such as difficult maintenance and rigidity of cable-based (non wireless) systems [38].

IoT systems consist of the Perception layer, the Network layer, the Middle-ware layer and the Application layer [38]. The Perception layer consists of multiple IoT devices that collect data, which is then sent via the Network layer to the Middle-ware layer. Here, the data can be stored and analyzed before it is processed for the user in the Application layer.

One of the most important technologies which is the basis of the IoT is wireless communication technology. Several wireless communication standards for different application profiles have been developed. While some, as, e.g., Wi-Fi offer very high data rates, they are less suitable for battery powered setups where low current consumption and at the same time large communication range is required (other systems such as GSM or satellite communications are less likely to be employed due to their comparably high costs [39]). Systems like Bluetooth offer low energy consumption (Bluetooth Low Energy), but are restricted in terms of their range. The LoRa technology is one of numerous low power wide area networks (LPWANs) which offer very low power consumption, while at the same time providing a wide coverage of 2–5 km in urban and 10–15 km in rural areas refs. [40,41,42]. For landslide monitoring, LoRa has several advantages over similar LPWANs, for example a payload size which is sufficient for data from multiple sensors on one sensor node (max. 243 bytes) [41]. Furthermore, it is bidirectional, which allows a degree of control over the sensors from the application layer (e.g., changing the time between measurements or updating firmware). Alternatives to LoRa are, e.g., Sigfox and NB-IoT, but LoRa offers the best parameters for this application: it is unlicensed (which NB-IoT is not) and bidirectional (which Sigfox is not), both important factors for a flexible, open-source system [41]. LoRa is being used worldwide in various natural hazard scenarios [39,43,44]. Other landslide early warning systems use or have used similar LPWAN technologies [45,46].

A LoRa data transmission is divided into uplink (device to gateway) and downlink (gateway to device). The uplink usually is significantly bigger than the optional downlink. The uplink packet size has a theoretical maximum of 255 bytes, depending, among others, on the distance from the device to the gateway [39]. In practice, the packet size should not exceed 51 bytes [40]. The LoRaWAN protocol restricts the maximum airtime of devices to 30 s per day, resulting in 20–500 messages per day, depending again on the distance/data rate and the payload size. This ensures that many devices can be in the same network without problems.

Another important technology, which has made the IoT possible, is the development of MEMS (micro-electro-mechanical system) sensors. These combine micro-mechanical elements and electronics in a single chip, allowing to develop small, highly available and low cost sensors for different measurement tasks. MEMS-based sensor systems already are being widely used for geotechnical instrumentation and landslide monitoring, especially since open-source microprocessors have become readily available in the last years [31,43,44,45,46,47,48,49,50,51,52,53,54,55,56,57]. They can be a good addition to classic monitoring methods, as discussed by Cmielewski et al. (2013) who investigated the accuracy and precision of a low-cost MEMS accelerometer [53]. They have been in use for rockfall monitoring [43,58,59], but also for shallow rotational landslides. Dikshit et al. (2018) developed a MEMS-based subsurface sensor with a volumetric water content sensor and a tiltmeter [60]. This concept, which is based on that proposed by Uchimura et al. (2010) [61] has also been applied for other LEWS/sites [52,54,62].

## 3. Inform@Risk Monitoring System

As stated before (Section 1.3), the geology of the project area is quite heterogeneous and complex, while at the same time a high landslide risk is present. With traditional measurement systems, monitoring this area is not possible, especially since it is an informal settlement where cost is one of the most important factors.

Therefore, we developed a LEWS which bridges the gap between local and territorial systems. It offers comparably high spatial and temporal resolution while at the same time being able to monitor a whole neighbourhood where the exact failure location is unknown. On the other hand, it is capable of issuing reliable site specific early warnings without previously knowing where a landslide will develop. Additionally, the system should be low in cost and should be designed specifically for the use in urbanized low-income areas. Existing monitoring technology—including promising remote sensing techniques as, e.g., SB/GB D-InSAR, laser scanning and drone based photogrammetry—cannot achieve this, because they do not have the required temporal or spatial resolution, are not reliable enough during bad weather conditions, are to complex in operation or are too expensive.

To meet these goals, the EWS requires a network of geosensors throughout the area, which in general are statistically distributed. The sensor density thereby is varied based on the landslide risk. This approach improves the benefit-cost ratio of the system, since in areas with low landslide risk, less sensors are distributed and therefore the density in high-risk areas can be increased. Hence, prior to planning the system, at least a qualitative risk assessment should be performed for a successful and effective system.

In this chapter, the newly developed sensor systems are presented. First, we give an overview of the general layout of the EWS which comprises a combination of traditional landslide monitoring systems as well as new geosensors which use IoT technology. The overall system has been presented before in preliminary studies [20,63,64] but here we give a more detailed and in-depth description of the newly developed IoT devices (LoRa nodes).

### 3.1. Monitoring System Layout

The LEWS mainly consists of deformation measurement-systems installed on the surface (e.g., attached to existing infrastructure), shallow subsurface up to about 1 m depth (e.g., inclination sensor probes and linear extension and shear measurement systems placed in trenches) and at some points deep subsurface in drillings. The system is based on the following sensors/measurement devices:

1. Horizontal Continuous Shear Monitor (CSM) measurement lines and extensometer (EXT);
2. Wireless LoRa sensor nodes;
3. Piezometers, extensometers and vertical CSM in drillings.

**Figure 1:**
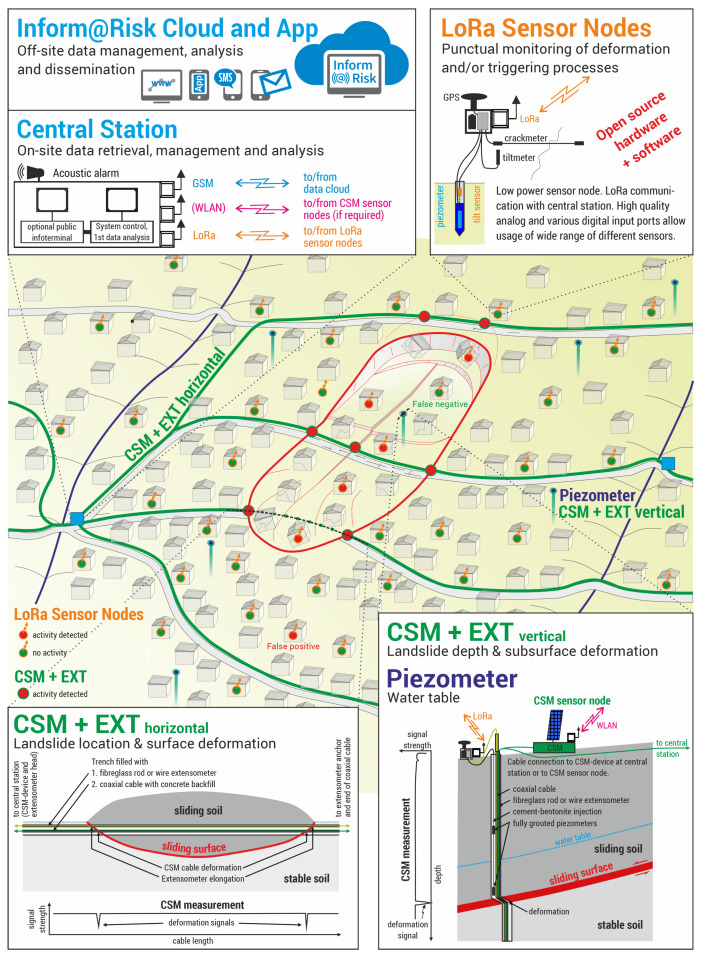
Schematic layout of the Inform@Risk monitoring system [64]. Data from CSM (Continuous Shear Monitor) and extensometer (bottom), as well as LoRa (Long Range) Nodes (top right) are combined in the Inform@Risk network and cloud system (top left).

These devices as well as the gateways and data management system are shown in a schematic layout of the whole EWS in Figure 1.

The horizontal and vertical CSM lines provide spatially and temporally highly resolved measurements of shear deformation along coaxial measurement cables. The cables are installed horizontally across the slope in trenches (ideally on multiple levels throughout the slope) and vertically in boreholes, so that possible landslide movement will be oriented orthogonally to the cable axis, thus shearing the cable [27,65]. If shear deformation occurs, the CSM measurements allow to detect the exact location of deformation and to estimate the amount of deformation [27,65]. The wire-extensometers which are installed parallel to CSM provide additional extension measurements, which allow to observe large deformation amounts in the dm to m range. While the CSM and EXT provide seamless observations along the measurement lines, their application is limited by the required free space and the laborious installation procedure. To fill the gaps between the CSM and EXT lines and to make the implementation of a wide range of sensors possible, the system is complemented by LoRa sensor nodes in different setups. They all share the same basic design but differ by their placement and the number and types of sensors attached to them. As shown in Figure 1, there should be at least two LoRa gateways in the system because of redundancy. Ideally, these gateways are positioned at points where the horizontal CSM lines intersect and/or where drillings are located. This makes it possible for the different measurements systems to share the required infrastrucure (e.g., enclosure, power, data connection) with the LoRa gateways. The connection from the gateways to the internet (“Network-” to “Middle-ware” -layer) is achieved using GSM modems and/or DSL via landline.

In the following, as one of the most important new developments of the Inform@Risk project, the LoRa nodes are described in detail [20,66].

### 3.2. Measurement Concept for the LoRa Sensor Nodes

A modular design has been chosen for the Inform@Risk LoRa sensor nodes. While all nodes share the same central hardware, as, e.g., the microprocessor, some basic sensors and the LoRa communication hardware (Inform@Risk LoRa base module), additional components can be added depending on the requirements of the specific monitoring task and installation location. This especially applies for the power supply (solar, battery and external power source) and optional sensor attachments. While the LoRa base module in general offers the flexibility to attach a wide range of different sensors, two specific sensor attachments have been designed for the use in the Inform@Risk LEWS. As a consequence, three types of sensor nodes can be distinguished which mainly build up the LoRa geosensor network of the LEWS: the Infrastructure Node (IN), the Subsurface Node (SN) and the Low-Cost Chain Inclinometer (LCI). While the IN only consists of the base module (with the option to attach further sensors based on need), the SN and the LCI have specifically designed sensor attachments. Each type of node serves a different measurement purpose: The IN is designed to be attached directly to existing infrastructure on site (e.g., buildings, posts, retention walls) and to monitor their movement/deformation. This is mainly done based on high accuracy inclination measurements utilizing an integrated MEMS sensor. When needed, additional sensors such as crackmeters or extensometers can be added to improve the quality of observation.

The LCI and SN both are designed to monitor subsurface deformation and ground- or seepage-water levels. While the LCI is based on the principle of inclinometer measurements and allows to quantify horizontal displacement, the SN only delivers a qualitative indication and possibly a semi-quantitative assessment of deformation through time. On the other hand the SN allows to perform highly resolved open pipe groundwater-level measurements based on a buoyancy rod, while the LCI utilizes a series of float switches usually with 0.5 or 1 m distance, thus delivering a much lower resolved information on groundwater levels.

**Figure 2:**
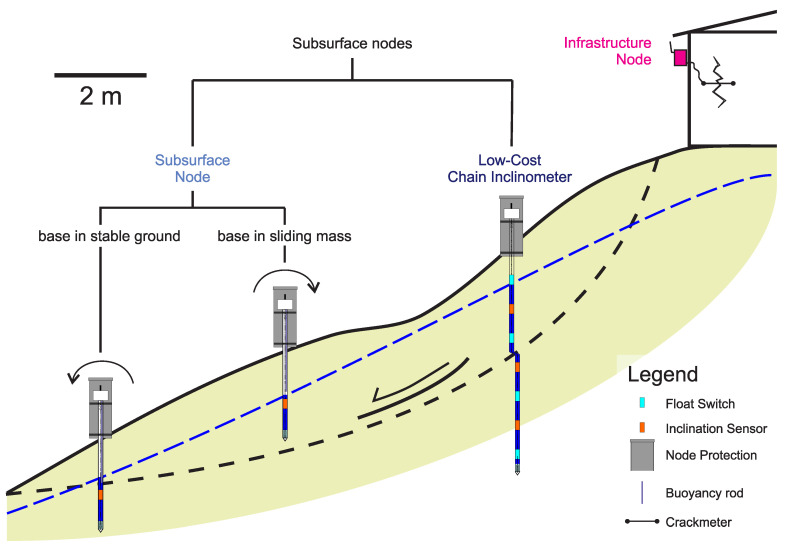
Measurement concept for the Infrastructure node (IN), Subsurface Node (SN) and Low-Cost Chain Inclinometer (LCI).

As the requirement for the successful implementation of a LCI sensor probe is to penetrate the basal shear surface of a landslide, these probes have to be installed into greater depths than the SN. The LCI consists of a series of inclination sensors (0.5 or 1 m interval) installed into an easily deformable PVC pipe, while the SN only has one inclination sensor and is installed into a stiff steel pipe. As the LCI penetrates through the sliding mass and the bottom lies in the stable subsurface, the tilt observed by the chain of inclination sensors can be used to calculate a profile of horizontal displacement throughout the subsurface, allowing to determine the depth and amount of deformation in the landslide. Provided the movement is a rotational slide, the SN will tilt upwards if it ”floats” in the sliding mass, and tilt downwards if the SN reaches stable ground (Figure 2). As the exact geometry of the landslide is unknown, SN measurements can only semi-quantitatively monitor landslide movement. These mechanics are also described by Qiao et al. (2020) who explored the direction and pre-failure tilting behavior of slopes and how this affects measured tilting angles of steel rods of different sizes [60,67].

As stated earlier, the sensor placement should generally be adapted according to the previously performed risk analysis. Additionally, it is important that each individual sensor location is checked before the installation to ensure that the sensor will be able to detect and measure a possible landslide. Therefore, it is also compulsory that each node location and installation is documented in detail, so it is possible to interpret the data in a meaningful way. In order to facilitate this process, we plan to add an interactive sensor installation guide and documentation feature into the Inform@Risk app.

### 3.3. Inform@Risk LoRa Base Module

**Figure 3:**
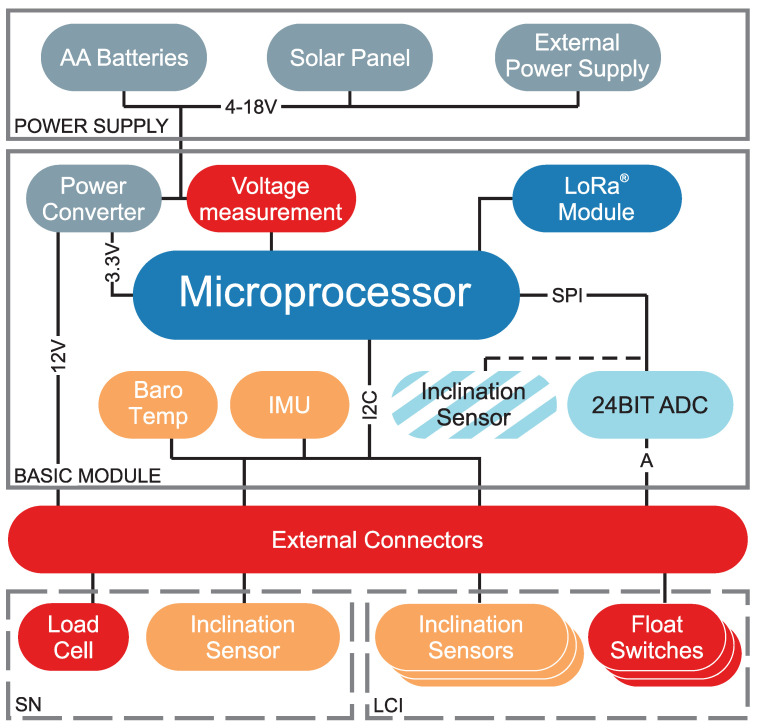
Schematic depiction of the Inform@Risk basic module and the additional sensors for subsurface measurements (SN, LCI). The colors show the data transmission: red: analog signal; orange: I2C (Inter-Integrated Circuit) bus; violet: SPI (Serial Peripheral Interface) bus.

The Inform@Risk LoRa base module is designed around a SAMD21 32-bit microprocessor board (Arduino MKR WAN 1310) with an integrated LoRa module (Murata CMWX1ZZABZ). This processor board has the benefit of combining very low power consumption with good flexibility in terms of analog and digital in- and outputs. Furthermore, its design is open-source and it is easily available all over the world. Figure 3 shows the basic design with the attached sensors (and the optional power- and sensor attachments). The detailed circuit board design, schematics and hardware specifications are referenced in Section 6. For easy reproducibility and further development a breadboard-based design is available. For actual applications it is recommended to use printed circuit boards (PCB) which make node construction much easier. The PCB and the breadboard-based designs are available for download on our website (Section 6).

Three different power supplies can be used: battery power (4–8 AA batteries), solar power (6V solar panel, 1–2 W, LiPo battery and battery charging regulator) or an outside power connection (DC 4–18 V input, optional with LiPo battery). All these options are connected to a voltage regulator, which supplies the required DC 3.3V internal system voltage. Additionally a step-up/down converter offers switchable DC 12 V for, e.g., external 4–20 mA devices. If batteries are used, an input voltage of about 6 to 12 V, corresponding to 4 to 8 AA batteries in series, is recommended.

The base module includes a low-cost IMU (Inertial Measurement Unit), which measures the acceleration (0.04° precision) and orientation of the node, as well as temperature and barometric pressure. Additionally, all Infrastructure Nodes include a high-precision inclination sensor. Because of its price, this unit is optional for all subsurface sensor nodes, where the inclination of the basic module itself is not as important. The sensor can simply be plugged into the circuit board when needed. We also developed a 3D-printed mount which allows the sensor to be placed horizontally, independent of the placement of the node enclosure (e.g., on a skew retaining wall).

Furthermore, included on all nodes is a 24-bit analog-digital converter (Texas Instruments ADS1220), to which external geotechnical or other sensors can be attached. It has four channels, which can be combined to max. two differential voltage measurements (e.g., for potentiometers) or can individually measure voltage referenced to ground (used, e.g., in combination with a measurement resistors for 4–20 mA measurements).

The firmware for the nodes is written in the Arduino programming environment (Integrated Development Environment, IDE) and can easily be accessed and changed with the IDE. The code is distributed via Github (see Section 6).

The software comprises five stages: initialization of the sensors, measurements, computation, and LoRa up- and downlink. Although these stages are fixed, multiple parameters can be changed to accommodate for varying on-site requirements. For example, the overall measurement duration as well as the measurement frequency for each sensor can be changed. These parameters can not only be changed when installing the node but also remotely with commands transmitted via LoRa communication. Furthermore, for each sensor it is possible to decide whether median or mean values should be sent to the gateway. For example for the accelerometer, it is best to calculate the median of the measured values since it is less sensitive to outliers from external influences (e.g., vibrations, impacts). For all other values, usually mean values can be chosen. The calculation is done by the microprocessor to save power and on-air time during LoRa communication, which uses the most power. With an average sleep interval of 15 min, the measured lifetime should be at least two years for the basic infrastructure node with six AA batteries (daily power consumption at 3.3 V is ca. 5–6 mAh).

After the measurement, calculations and LoRa-uplink, the device receives an optional downlink from the gateway. This can be one of several predefined commands such as setting the looptime (time taken for measurements and time the device is asleep), changing measurement duration or activating/deactivating individual sensors on the device. After receiving commands, the device makes the changes and goes into a sleep mode, in which current consumption is minimized. More information on the firmware can be found in the documentation provided on the Github page (Section 6).

### 3.4. LoRa Sensor Nodes

#### 3.4.1. Infrastructure Node (IN)

The IN is the base module usually with the optional internal high quality inclination sensor installed. This sensor (Murata SCL3300) has a precision of 0.003°, and thereby, even very slight changes in inclination can be measured (e.g., if a 1 m high retaining wall tilts by more than 0.1 mm, this movement should be detected). The PCB is placed in a waterproof housing, still allowing optional sensors to be attached, as stated earlier.

#### 3.4.2. Subsurface Node (SN)

**Figure 4:**
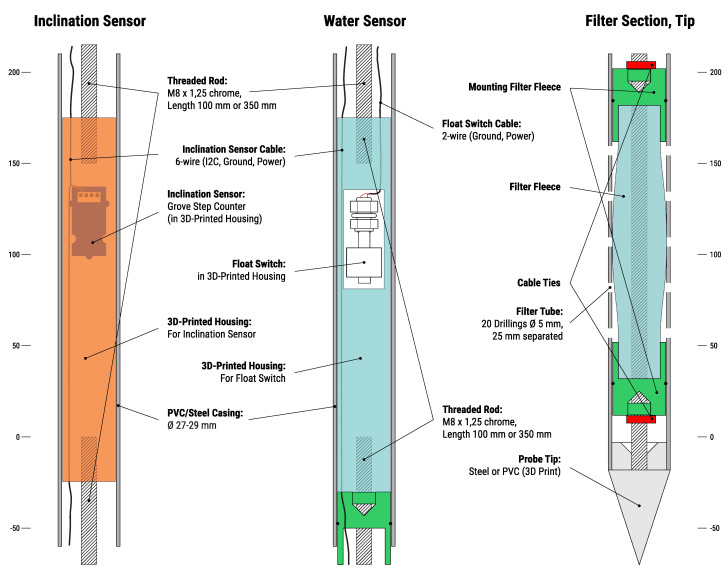
Hardware design of the inclination sensor, the groundwater sensor and the tip and filter section for both the SN and LCI.

The SN and LCI are both subsurface attachments to the base module. After they are installed in the subsurface on site, the base module can simply be installed on top of the housing with a simple 3D-printed adapter (see Section 6). Inside the rod of the SN, one inclination sensor is included to be able to detect tilting of the rod. The casing of the rod can be made of PVC or stainless steel, but steel casings are preferable since they can be installed without pre-drilling. The detailed sensor- and connection-layout is shown in Figure 4.

The inclination measurement is performed by a triaxial accelerometer (BMA456, 0.02° precision). It is connected to the basic node via the I2C bus. To house this sensor, a 3D-printed housing has been developed. It provides safety for the sensor, as well as a steady fixture for accurate readings. The sensor unit itself and the cable connections are waterproofed with a 2-component sealing gel. The SN is usually installed into depths of up to about 2 m.

The groundwater measurement is performed using a load cell together with a buoyancy rod made of PVC, which allows for continuous measurement, but only in the top part above the inclination sensor, depending on the length of the rod.

#### 3.4.3. Low-Cost Chain Inclinometer (LCI)

The LCI is similar to the SN but includes inclination sensors every m, resulting in up to 6 inclination measurements in the subsurface. They are all connected in serial to the I2C bus. Each of them is wired to a different digital port or addressed differently on the I2C bus, respectively. In contrast to the SN, the casing should be made of PVC in order to allow for deformation of the rod to determine the shear depth. The inclination sensor is the same as for the SN. Currently due to limitations of the used digital bus (I2C) the max. achievable depth for the LCI is about 6 m. The groundwater measurement is achieved with a float switch each m, alternating with the inclination sensor. They act as a series of resistors in parallel, which increase the resistance with increasing groundwater level. They are connected to the analog input of the microprocessor or the ADC. Therefore, the groundwater measurement of the LCI is not as good as the one the SN provides, since it is not continuous. Still, the most important information—whether there is water in the top meters—can be gained this way.

### 3.5. Installation

**Figure 5:**
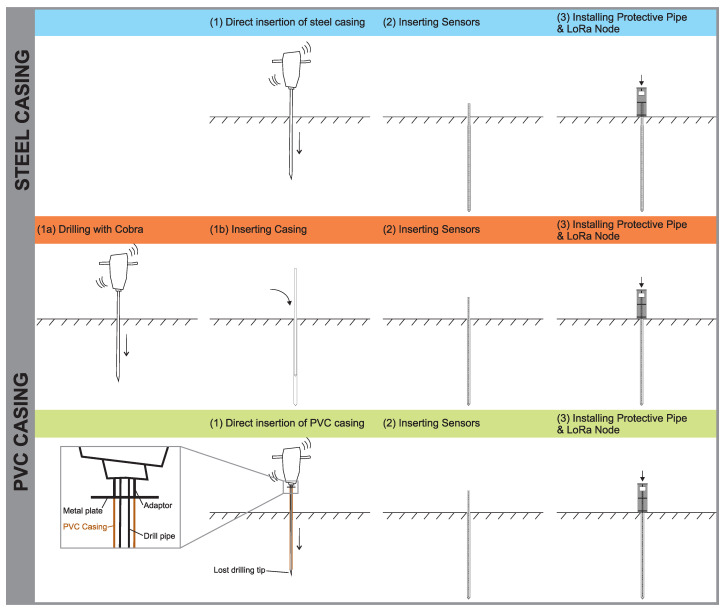
Installation suggestions for steel casing (blue) and PVC casing (red, green).

In general, the installation of the subsurface probes (SN and LCI) can be done with three different techniques, as shown in Figure 5. The first option is direct insertion of the steel casing, using a hydraulic, pneumatic or petrol jackhammer or heavy dynamic probing equipment (DPH). The second option is to pre-drill a borehole of 45–50 mm using a drilling method fitting to the geological conditions and afterwards insert the casing (steel or PVC pipe). The third option allows to directly insert a PVC pipe into the ground using a jackhammer or DPH equipment. This is achieved by placing the steel drilling rod inside the pvc pipe and using a “lost” metal tip, which is only loosely attached to the drilling rod and stays in the ground when the drilling is completed and the drilling rod is retracted. To ensure the PVC pipe is is pushed into the ground simultaneously with the drilling rod, a small metal plate with a diameter larger than the pipe is attached to the top of the drilling rod.

Which installation technique can be used mainly depends on the subsurface material, the depth and the probe type to be installed. For the SN usually drilling method 1 and for the LCI drilling method 3 will be the best option, as long as no hard rock (stones/blocks or bedrock) is encountered. If this is the case, drilling method 2 will have to be used, which—depending on the required drilling method—makes the installation more complex and costly. This limitation is more significant for the LCI, as it requires higher drilling depths.

After the insertion of the casing, the sensors, which are prefactured on the threaded rods (see Figure 4) to lengths of 1 m are installed in the casing and connections are made between each m-segment during the installation. Once all sensors are inserted in the borehole, the LoRa node itself is attached to the top of the casing and the subsurface sensors are connected to the node. Depending on the situation, a protective pipe or some other kind of protection should be added over the node.

**Figure 6:**
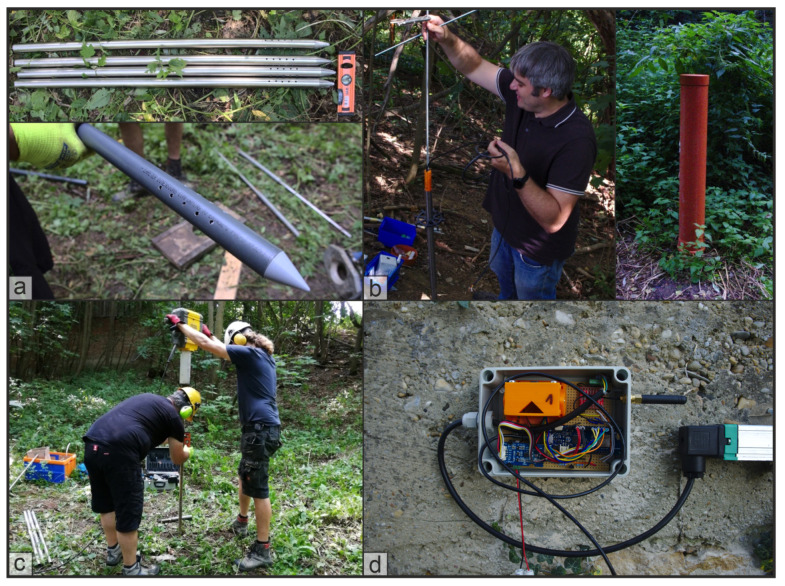
Pictures from a field installation where the presented sensors were tested and evaluated and the installation procedures were developed. (**a**) Steel and PVC housings with drilled holes for the filter section. (**b**) Left: installation of sensors into the housing, right: preliminary sensor encasing. (**c**) Installation of the housing with a jackhammer. (**d**) Preliminary circuit board for the IN on a wall with attached potentiometer.

The installation of these sensors has been tested on a field site in southern Germany (see Section 3.6). Here, also different sensors and attachments were tested in order to find the now final sensor layout. Pictures of this test installation are shown in Figure 6.

As stated before, the main goal of the system is to increase availability by keeping the costs to a minimum, where possible. The LoRa base module costs about 100 €, including all electronics and power supply. Depending on the attachments, the IN then costs on average 50 € more and the SN and LCI are approximately 100–150 € more expensive because of the casings and the subsurface sensors. The work hours are not taken into account and we are aware that this is also an important cost factor. Yet, it is hard to determine these costs and also, it has been our experience that the local residents are often very willing to help, and they gain more trust to the system if they are engaged in the installation process. If the Inform@Risk PCB is used, the assembly should take about 15–30 min per node, with an additional 30 min for the SN and LCI. The installation on site varies from about 30 min (IN) to 2–4 h for the SN and 4–6 h for the LCI (including drilling), of course depending on the geology and other conditions on site.

Comparing these costs with a regular chain inclinometer (starting from approx. 5000 € excl. drilling), our system is more cost effective, although it is clear that the same data quality cannot be achieved. While a chain inclinometer produces absolute deformation measurements with very high accuracy and precision, the SN only provides semi-quantitative data, as we only measure the tilting of the top layer. The LCI on the other hand should provide absolute inclination data, but is limited in its depth and its setup is less robust than a regular chain inclinometer due to the 3D-printed housings and threaded rod system. The most important factors about the developed LoRa sensor nodes are summarized in Table 1.

### 3.6. Test Site Near Regensburg, Germany

**Figure 7:**
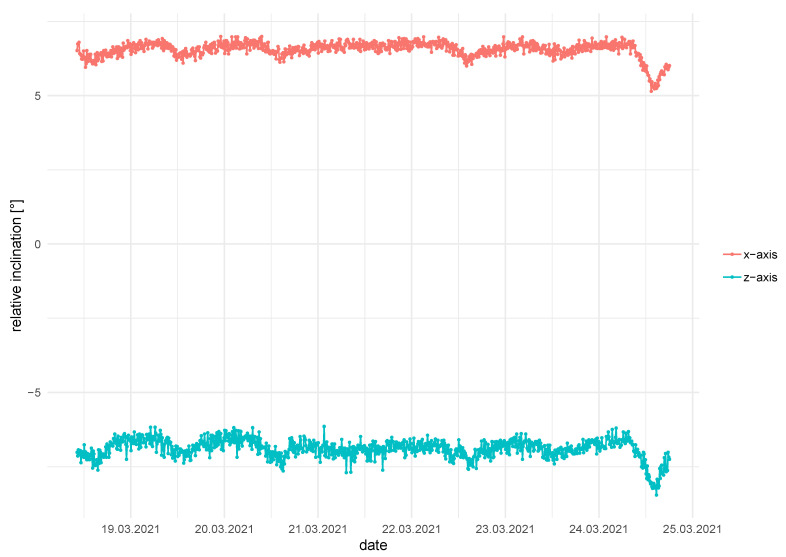
Exemplary inclination data taken from an accelerometer on top of a subsurface node. While the measurements do not yet show deformations, daily cycles as well as sensor noise are visible. The sensor noise in this real-world application is higher than that measured in the laboratory.

To test these LoRa nodes, we created a number of prototypes in order to test the functionality and longevity in a field installation. Because of the pandemic, it was not possible to do this in Colombia, so we chose a site in southern Germany near Regensburg, which has similar properties. A rotational landslide in fine-grained material (sand, silt and clay in varying proportions) started to develop some years ago and is slowly moving towards railroad tracks. This slope is already being monitored by two inclinometers. In parallel to these, we have installed six of the Inform@Risk LoRa nodes: Two IN on a wall (Figure 6d) and four variations of the SN and LCI in different depths and sensor constellations. The data are being collected by a LoRa gateway which is about 100 m from the sensors. The sensors, which were installed in July 2020, show a robust and continuous data transmission. However, no inclination measurements could be detected right now which could be confirmed by the inclinometer measurements. This installation helped us in improving the hardware, software and installation procedure to be more reliable. In Figure 7, the inclination data of the IMU in one node is shown as an example. Although only temperature cycles are visible, they show that even the low-cost sensor is capable of replicating these slight changes in inclination.

While the presented subsurface sensors offer an effective method of directly measuring subsurface deformations, it must be emphasized that they are mainly applicable to shallow rotational landslides. Especially the SN might not detect the movement if other landslide mechanisms (e.g., purely translational sliding) are present. In general the application of the proposed sensors has to be critically checked based on the geological process and hazard analysis. While the presented systems have been tested for about half a year now, still further testing is required to prove the full functionality of the system. This includes tests to determine the sensitivity of the SN for different geometrical setups and direct comparisons of LCI measurements with classic inclinometer measurements.

## 4. Data Processing

All data acquired in the geosensor network is immediately transferred to an off-site central server (Inform@Risk cloud), where it is processed and analyzed in near real time.

### 4.1. Data Transmission and Storage

**Figure 8:**
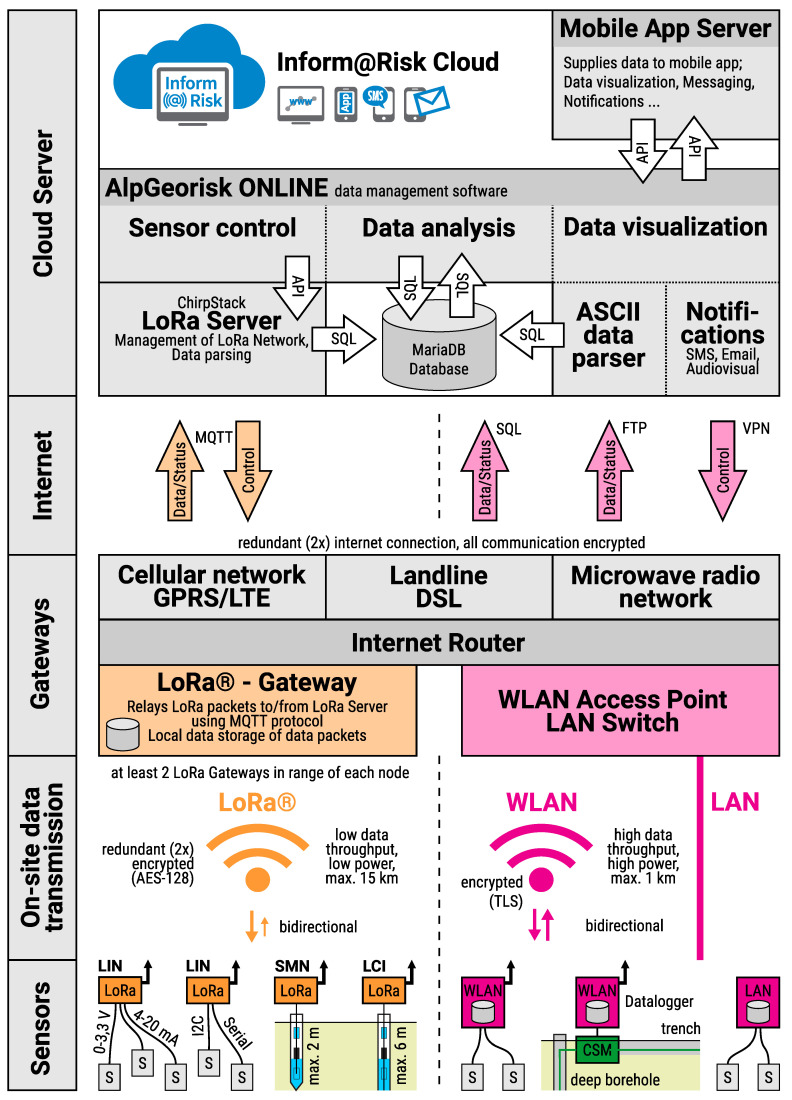
Data processing concept of the Inform@Risk project.

In case of the LoRa geosensor network the encrypted data packets sent from the nodes are relayed through the LoRa gateways on site to an off-site LoRa Network Server server using a redundant internet connection via DSL, Cellular network and/or microwave radio (Figure 8). The LoRa server manages the LoRa communication and relays the data to the LoRa application server, which then analyses and decodes the encrpypted data packets and writes the data into the Inform@Risk cloud database. The LoRa server is also responsible for sending control commands to the LoRa nodes via the most appropriate gateway. For the inform@risk project the open-source LoRa network server “Chirpstack” (www.chirpstack.io) (accessed on 1 March 2021) is used.

As the CSM measurements produce a large amount of data (up to about 350 kB uncompressed data per measurement), LoRa can not be used to transmit the CSM data. Therefore, the system is designed so that, whenever possible, the CSM measurement devices are located at the LoRa-gateways. This allows the CSM devices to directly utilize the internet connection of the gateways to transfer the data to the Inform@Risk cloud. Whenever this is not possible, local wireless LAN connections with directional antennas are used to relay the data to the gateways. All gateways are equipped with backup batteries (200 Ah) which allow them to operate for two weeks without external power in case of an emergency. An open source MariaDB database is used as central data storage. In general, the raw data received from the sensors is stored and kept unchanged in the database. Any further processing (see below) of the data is stored at different locations in the database, making it possible to change calibration and analysis parameters afterwards if deemed necessary.

### 4.2. Data Management

In the Inform@Risk project the AlpGeorisk ONLINE (www.alpgeorisk.com) (accessed on 1 March 2021) data management platform is used to manage the large amounts of data (several GB per month) which are generated by the geosensor network. This platform is responsible for data quality management (identification of data errors), calibration (conversion of raw data to measurement data), storage (structured storage in a relational database), analysis (see below) and visualization via web-portal as well as system management (sensor status checks) and control (e.g., change acquisition interval) and notifications (alarms and early warnings via email, SMS and/or sirens etc.). As part of the Inform@Risk project, a mobile app is being developed to complement the AlpGeorisk ONLINE data management system and make the data more accessible and understandable for the residents in informal settlements. The presented monitoring system can of course also be used in combination with a different data management platform (e.g., if one is already available), but the combination with the AlpGeorisk system is very convenient since it is already adapted to the specific requirements.

### 4.3. Data Analysis

The data analysis methodology being developed for the Inform@Risk LEWS is based on a combination of dynamic thresholds for trigger- and deformation observations, sensor fusion methods and pattern recognition methods. As the geosensor network is designed for densely populated areas, accidental or intentional tampering with the sensors has to be taken into account. Otherwise, a large number of false alarms would be the consequence—which, even if reviewed by an expert before issuance of a notification, would most likely make an effective operation of the system impossible. For this reason we plan to apply sensor fusion and pattern recognition techniques to filter the incoming data and separate the significant from the insignificant data. For example, when a threshold is surpassed on a single inclination sensor, the system checks if any of the surrounding sensor nodes have also registered a deformation increase. It also checks if any of the sensors monitoring the triggering factors (rainfall, groundwater, seismic events etc.) show an increased level. Depending on the severity and degree of confidence of the observation, either the event is temporarily ignored or appropriate action is taken, which ranges from marking the event for review through the system manager to issuing an alarm. In any case, the system will instruct the relevant sensors to increase the measurement interval and as further data of the event is collected throughout time, the system will reevaluate the event utilizing pattern recognition methods on the time series of the relevant deformation and triggering datasets. This cycle is continued until the event is identified as an outside influence, or—if the situation remains uncertain—is reviewed and classified by the system manager. With time, the system will collect a database of classified events, which can be reviewed periodically and serve as training data for the pattern recognition algorithms. The thresholds used for the different sensor types at the different locations are first determined based on sensitivity analyses performed using the geological/geomechanical models created during the hazard analysis and include absolute values as well as rates where applicable (e.g., deformation rate). Later with increasing length of observation the thresholds are adapted using the results from timeseries-analyses performed on the collected data. This also includes trigger informations, especially for rainfall, one of the most important trigger factors. The data analysis methodology for the Inform@Risk LEWS outlined above is currently still in development. Its functionality will be evaluated and improved during a planned “learning-phase” of the LEWS of at least one year.

### 4.4. Data Dissemination

Based on the analysis results, the short- to medium-term hazard level is assessed, and—if deformation is detected—early warnings and alarms are issued. The main information dissemination tool will be a newly developed mobile app.

#### 4.4.1. Short- to Medium Term Hazard Level

In order to assess the short- to medium-term hazard level, mainly the triggering factors rainfall and groundwater height are considered. On the one hand, time series analyses will be performed to identify causal and temporal relationships between short-, medium- and long-term rainfall and groundwater levels [68]. On the other hand, the hazard level is determined using groundwater level thresholds at, e.g., 50, 75 and 90 percent of the critical water table derived from the geological/geomechanical models created during the hazard analysis. The threshold values thereby are determined individually for different geological homogeneous zones throughout the project area and are applied to the according sensors in this area.

#### 4.4.2. Early Warning and Alarms

Early warnings are issued only if significant deformation has been detected. Depending on the amount of deformation observed, different early warning levels are issued. The number and value of thresholds used to define these levels are currently being determined, but early warning will most probably cover the range from mm per year up to cm or dm per hour. Based on how many and which neighbouring sensor nodes show deformation, the affected area and landslide mass are estimated and reported. If a further or sudden strong acceleration is detected, the system can issue an immediate (evacuation)-alarm using acoustic and/or light signals. Another factor that is important for the early warning are measurements of the trigger parameters, most importantly rainfall. It is measured on site by a weather station and a rainfall radar of the city of Medellín is used for forecasts. To make the connection between rainfall and groundwater levels, a hydrogeological model is used and combined with the data gathered during the test phase of the system. This test phase serves to make the system more accurate for predicting the influence of rainfall on groundwater levels, and therefore, hazard levels. Depending on the hazard state, deformation rate and the affected area different actors (experts, trained community members, first responders, whole population) are informed. Usually warnings will be checked by an expert before they are sent to the inhabitants. Only when at least two neighboring sensor nodes show very strong acceleration at the same time and the data has successfully been crosschecked according to the above data analysis procedure, the warnings are issued without review. Right now, we plan on warning the inhabitants using the Inform@Risk App, as well as multiple sirens which will be installed in the area. The exact definition of the warning levels, warning contents and the information dissemination paths are being developed in a participatory process, ensuring that, if possible, each actor gets the required information at the right point in time.

## 5. Summary and Outlook

A new IoT based geosensor network for communities in informal settlements has been developed, mainly based on open-source hardware and software. The sensor network consists of three different sensor nodes, which allow to monitor movement/deformation of the subsurface (SN, LCI) or existing infrastructure (IN) as well as the groundwater conditions in near surface soil layers. The subsurface sensors operate most efficiently for shallow rotational landslides. If translational or deep seated landslides are expected, the effectiveness of the system is reduced. In general, the applicability and placement of each sensor node has to be evaluated based on the expected landslide processes and vulnerability to outside influences.

While the newly developed sensor nodes are not as precise as existing high quality geotechnical sensors for landslide monitoring, they offer reasonable measurement quality at much lower cost. Thus, they can be distributed in comparably high numbers over an area, whereby sensor density is varied depending on the hazard level and the expected landslide mechanism. This leads to the necessary spatial density of observations, which is required to reliably detect the onset of landslide movements anywhere in the monitored area. When additionally complemented with other monitoring techniques as, e.g., the Continuous Shear Monitor (CSM) and sensors observing the triggering factors, a versatile, robust and flexible LEWS can be created.

A limitation of the system can be expected when very small landslides occur. Since the scenario this system aims at is quite difficult (shallow landslide at unknown location), it is possible that a small landslide occurs in between the sensors. This risk depends on the quality of the hazard and risk assessment and the density of CSM lines and LoRa sensor nodes. Bigger landslides should easily be detected by this system, except for very large and slow creeping which does not cause noticeable shearing and tilting on the surface. These slow movements, on the other hand, are not as relevant to this system because they pose less risk to the residents’ lives. Another possible drawback of this system might insufficient pre-failure deformation before the event. If there are no significant shear- or tilt-deformations on the surface before an event, it is not possible to issue a warning based on deformation data. This is especially the case with the shallow measurements this system is based on. Therefore, the system needs to incorporate trigger data and it is incessant to have a learning phase where the thresholds for both trigger and deformation data can be defined and evaluated.

The large amount of data produced by the system and the high number of outside influences make new data processing procedures necessary, in order to ensure an effective operation and prevent false alarms. These procedures have been outlined here and will be presented in more detail in a future contribution after the system has been implemented and tested. Especially the relation between weather forecasts, groundwater levels and deformation measurements will be addressed in more detail.

As already stated, currently further tests of the sensor system are carried out in Germany. As soon as the COVID-19 related restrictions allow for it, the first installation of the proposed LEWS will be performed in Colombia. This system will comprise about 130 Sensor nodes, five drillings with instrumentation and about 1.5 km of horizontal CSM/EXT lines. The installation will also be used to gain further experiences in terms of the social integration of the system. Concepts as, e.g., ’sensor god-fatherhood’, meaning that if, e.g., a sensor node is installed on a house wall, the house will take care of the sensor node, will be tested. After an operation of about one year the complete system will be evaluated and recommendations for future developments stated.

All new findings of the Inform@Risk project are being developed as open source and will be distributed freely. Hopefully this work can encourage researchers and risk managers throughout the world to implement and further develop comparable LEWS, especially in such areas of the world, which were often overlooked in the past. Please feel free to comment and actively contribute to the development on the provided project websites (see Section 6). Any criticism, suggestions or advice is welcome.

## 6. Resources

All hardware and software being developed in this project is open source and can be accessed and freely replicated (GPL-3.0).

The firmware for the sensor nodes can be accessed via our Github page:

https://github.com/moritzgamperl/informrisk-lora-node (accessed on 1 March 2021). Here, the firmware for the sensor nodes, the documentation, as well as the required libraries can be found.

The hardware specifications (schematics, circuit board files and documentation) and documentation for the individual sensors can be found on the following webpage, which will be updated regularly: www.informrisk.alpgeorisk.com (accessed on 1 March 2021). Here, also the files for 3D-printing can be accessed.

## Figures and Tables

**Table 1:** Overview of the LoRa sensor nodes, in comparison with a regular drilling and installation of a chain inclinometer. Installation: (++): easy, (+): intermediate, (-): difficult, (–): very difficult. RD: relative deformation measurements, AD: absolute deformation measurements, SQ: semi-quantitative measurements, GW: groundwater measurements.

Type	Depth [m]	Pipe	Installation	Data Quality	Cost
IN	-	-	++	RD; Wide range of sensors;	∼150 €
SN	<2.5	Steel	+	SQ (Tilt sensor); GW	∼200 €
LCI	3–6	PVC	-	AD (Tilt sensor); GW	∼250 €
Chain Inclinometer	>5	PVC/Alu	–	AD	>>5000 €

## Data Availability

As stated above, the sensor hardware and software developed in this study can be downloaded freely. Measurement data is available on request from the authors.
